# Supplementary material for: Efficacy of a randomized controlled trial of a perinatal adaptation of COS-P in promoting maternal sensitivity and mental wellbeing among women with psychosocial vulnerabilities
Source: PLoS One. 2022 Dec 1;17(12):e0277345. doi: 10.1371/journal.pone.0277345 (PMC9714844; doi:10.1371/journal.pone.0277345)
Supplement: S1 File — (PDF) [file pone.0277345.s002.pdf]

ClinicalTrials.gov PRS
Protocol Registration and Results System

ClinicalTrials.gov Protocol Registration and Results System (PRS) Receipt
Release Date: June 22, 2017

ClinicalTrials.gov ID: NCT03190707

Study Identification

Unique Protocol ID: CSU-2017-001

Brief Title: A Good Start to Life - an Early Cross-sectorial Intervention

Official Title: Early Prevention of Social Inequality in Health - an Interdisciplinary and Cross-sectorial Intervention for Vulnerable Pregnant Women

Secondary IDs:

Study Status

Record Verification: June 2017

Overall Status: Enrolling by invitation

Study Start: June 16, 2017 [Actual]

Primary Completion: June 30, 2019 [Anticipated]

Study Completion: June 30, 2019 [Anticipated]

Sponsor/Collaborators

Sponsor: Intersectoral Research Unit for Health Services

Responsible Party: Sponsor

Collaborators: University of Copenhagen

Oversight

U.S. FDA-regulated Drug: No

U.S. FDA-regulated Device: No

U.S. FDA IND/IDE: No

Human Subjects Review: Board Status: Approved
Approval Number: 17006186
Board Name: The Regional Committee on Health Research Ethics
Board Affiliation: The Danish Capital Region
Phone: 3866 6395
Email: margit.bom@regionh.dk
Address:

Kongens Vænge 2
3400 Hillerød
Denmark

Data Monitoring: Yes

Plan to Share IPD: No
Due to individual data protection we will not be able to share individual participant data (IPD).

Study Description

Brief Summary: The aim of the project is to develop and evaluate an interdisciplinary and cross-sectorial intervention targeting pregnant women with psychosocial vulnerabilities. The objective is to 1) detect depression, anxiety and personality disorders in the pregnant women, 2) to increase knowledge sharing across the health care sectors and 3) strengthen the parents' parenting skills and thereby support a secure attachment between parents and child and thus promoting the child's well-being.

The overriding hypothesis is that an early multi-stringed, interdisciplinary and cross-sectorial intervention, with a long-term perspective from the early pregnancy throughout the child's first years of life, can effectively prevent disorders in the parent-child relation. The approach is to detect and treat depression and anxiety in the mother and strengthen the parents' parenting skills in families with maternal psychosocial vulnerabilities. The hypothesis indicates that the over-all intervention will result in improved interaction between child and parents which will make it possible to detect higher maternal sensitivity and a higher level of well-being among both children and parents in the intervention group compared to the control group.

The projects' specific hypotheses are;

- A systematic screening for anxiety, depression and personality disorders in the midwifery consultation will imply more pregnant women with symptoms of anxiety, depression and personality disorders being detected and offered treatment in the intervention group compared to the control group.
- Knowledge sharing across health care sectors will improve by the implementation of a joint consultation involving the vulnerable pregnant woman/families, the midwife and the health visitor and by a systematic transmission of information when the woman leaves the post-natal ward and is transferred to the health visitor.
- Parental skills in psychosocial vulnerable pregnant women and their partners can be strengthened by a parental training program and by education and dialogue about perceived challenges and breast-feeding. Parents in the intervention group will therefore gain greater knowledge on what it takes to make breast-feeding work successfully, be able to interact more appropriate with their children, and improve their mentalization skills and experience less stress compared to parents in the control group.

Detailed Description: Design, material and method:

The project will be conducted as a randomized controlled trial. The intervention group will receive a specific intervention and the control group will receive care as usual, in this context the standard treatment in antenatal care for vulnerable pregnant women and by the health visitor.

Study setting:

The intervention will be executed on Herlev-Gentofte Hospital in Denmark and in four of the affiliated municipalities: Ballerup, Gentofte, Herlev and Rødovre.

Target group:

The intervention is targeted vulnerable pregnant women who do not have psychiatric diagnosis or problems related to drug abuse, are not treated at family outpatient clinic or comprehensive municipal precautionary interventions (care level 4*), but who has the need for more than the standard treatment due

to psychological and/or social challenges (e.g. previous traumas, problematic family relations or acute life crisis). Hence, the target group of the study is women with psychological or social vulnerabilities and classified as care level 3 by the hospital.

It is estimated that the project can identify 100 pregnant women from the target group from the four municipalities during the inclusion period of 10 months. An expected drop-out on 20 % will result in an expected amount of 80 pregnant women who can complete the project.

Recruitment:

Participants are recruited among pregnant women who are referred to give birth at Herlev-Gentofte Hospital. On the basis of information from the general practitioner, the referring midwife refers the pregnant women to one out of four care levels. From the above mentioned inclusion and exclusion criteria the pregnant women from care level 3* is identified as being in the target group by the referring midwife. Afterwards, the women are recruited to the project through an enclosed invitation and information leaflet, send from the hospital together with a notice for the first midwife consultation. Next, project personnel contact the pregnant women to consult whether they are interested to participate in the research project. Then, the project personnel visit the potential participants in their homes to give further information on the project and include the interested women and their families in the project.

Selection of intervention hospitals and municipalities:

An examination of existing or previously interventions targeting vulnerable pregnant women in the Capital Region of Denmark was utilized in the process of selecting hospitals and municipalities in the intervention and control group. Herlev-Gentofte Hospital was invited to participate in the project as these hospitals were suitable with regard to the projects key intervention components and already existing interventions on the hospitals and affiliated municipalities. The included municipalities confirmed that they are not participating in other research project in the field of early prevention of social inequality in health among vulnerable pregnant women as long as this project last. The municipalities are: Herlev, Ballerup, Rødovre and Gentofte.

Data collection:

Data for evaluating the efficiency of the intervention are collected on three points of time; at baseline, after birth, when the child is aged four weeks, and at follow-up after the intervention has finished, when the child is around nine months. All data are collected in the homes of the families. To make an extra incentive to complete the questionnaires the participants receive a gift to the value of approx. 300 dk. kr. (approx. 45 USD) when completion of the follow-up questionnaire. Baseline data is collected for both control and intervention group before participants are familiar with the allocation of the randomization. The following paragraphs will describe the type of data that will be collected. Table 4 shows an overview of variables expected to be collected. Two different questionnaires will be developed specifically for the mother and for the father. The questionnaires look alike except that the mother's questionnaire contains questions on her attachment to the child in the belly and on her attitude and perception of breast feeding.

The questionnaires will be pilot tested by individuals from the target group to ensure that the questionnaires have a manageable size for participants and that questions are relevant. Afterwards the questionnaire will be revised accordingly.

Planned statistical methods:

The analyses will be performed using the statistical software Statistical Analysis System (SAS) 9.4. Analysis and presentation of data will be in accordance

with the CONSORT guidelines (reference). Standard descriptive statistics (means, medians, ranges, standard deviations, frequencies and percentages) will be used to report demographics, baseline and outcome scores. Data will be examined for missing data and multiple imputation strategies will be used if necessary. It is expected that the frequency of missing data will be low as data are collected through home interviews.

The analysis of both primary and secondary endpoints will follow the intention-to-treat principles. However, analyses taking the actual treatment participation into account will also be conducted. To examine how non-compliance affects the results different levels of participation will be investigated.

Primary and secondary outcomes will be analyzed using multiple regression for continuous outcomes, and logistics regression for binary outcomes controlling for baseline scores when possible.

* * The Danish Health Authority's recommendations for antenatal care prescribe the general practice to classify all pregnant women in care level 1-4 based on a psychosocial anamnesis. Pregnant classified in care level 1 receives the standard intervention; pregnant in level 2 receives an extended intervention in the context on antenatal care based on e.g. previously birth complications or disease; pregnant in level 3 receives an extended intervention including involvement of other professional groups in the health care sector or municipality based on complex social, medical or psychological problems; and pregnant in level 4 receives an extensive interdisciplinary and intersectorial intervention, including collaboration with specialized institutions based on highly complex problems e.g. alcohol or drug abuse, severe mental/psychiatric diseases and/or severe social load

Conditions

Conditions: Anxiety
Attachment Disorder
Maternal Sensitivity
Depression
Personality Disorders

Keywords:

Study Design

Study Type: Interventional

Primary Purpose: Prevention

Study Phase: N/A

Interventional Study Model: Parallel Assignment
The study population will be stratified randomized on an individual level, thus half of the women, who agree to participate, is being randomized to the intervention group and the other half is being randomized to the control group. The trial participants will be allocated within each municipality to either control- or intervention group. The project personnel visiting the families to collect baseline data carry out the randomization. The project personnel bring a sealed envelope when visiting the families. In the envelope, the randomization group is stated together with a randomization number. The envelope is packed by a third party individual who receives a computer generated allocation list from another researcher. The envelopes get the numbers from this random sequence. The researcher generates the allocation list by block randomization with random

	block sizes of 2. The random allocation list is generated once and will be destroyed after the sealed envelopes are packed.
Number of Arms:	2
Masking:	Investigator, Outcomes Assessor
Allocation:	Randomized
Enrollment:	100 [Anticipated]

Arms and Interventions

Arms	Assigned Interventions
Experimental: Intervention<br>The intervention consists of three key components aiming at promoting a better attachment between child and mother/parents and through that giving the child the best possible beginning of life. The three components aim at: 1) detecting ill-being in vulnerable pregnant woman and initiation of potential treatment, 2) strengthening knowledge sharing and organizing the course for the families across the sectors, 3) strengthening parenting skills.	Behavioral: Intervention<br>1) Detection of ill-being: An extended consultation with the midwife. The midwife will screen the women for symptoms of anxiety, depression and personality disorders. 2) Knowledge sharing: An additional midwife consultation accompanied by the health visitor. The aim of is sharing knowledge and transmission of information between the two sectors responsible for making a shared plan for the course of the process for the pregnant woman. 3) Strengthen parenting skills through: - Two visits by the health visitor before birth and eight after. The visits are based on the parent training program The Circle of Security, aiming at promoting a secure attachment between child and carer. - Teaching the woman/couple on the importance of breast-feeding and how to succeed.
No Intervention: Control<br>The existing practice for psychosocial vulnerable pregnant women on Gentofte-Herlev Hospital, Denmark, will be offered for women allocated to the control group. The control group will be measured at the same follow-up periods as the intervention group.	

Outcome Measures

Primary Outcome Measure:

1. Maternal sensitivity

   Sensitive responses, that is the ability to respond appropriately to the child’s attachment needs, has consistently been found to be the most reliable predictor of attachment security. The intervention, including The Circle of Security, has the aim to promote maternal sensitivity.

   The measurement tool ”Coding Interactive Behaviour” (CIB) will be used to code maternal sensitivity towards the child. Maternal sensitivity is observed during 5 minutes interaction (free play) between mother and infant.

   CIB has good psychometric properties and has been validated in multiple longitudinal studies across cultures and across age groups (from newborn to adolescent). The system has previously been used for the purpose of research of the relation between a child and a mother with mental illness(es). Moreover, it has been used to evaluate the effectiveness of interventions.

   [Time Frame: The mother-child relation will be videotaped and assessed during a home-visit by the health visitor after the intervention is finished when the child is around 9 months old.]

Secondary Outcome Measure:

2. Prenatal Parental Reflective Functioning Questionnaire

   Measuring the mentalization skills or reflective functioning of the parents in relation to the unborn child.

   [Time Frame: Baseline]

3. Parental Reflective Functioning Questionnaire
   Measuring the mentalization skills or reflective functioning of the parents in relation to the child, and refers to the capacity to treat the child as a psychological human. Consists of three sub scales, describing high, low or nor high neither low mentalization functioning in the parents.

   [Time Frame: Second follow-up:15 months after baseline (child 9 months old)]

4. The Parenting Stress Index
   Measuring stress in relation to parents and in relation to the child and dysfunctionality in the parent-child relationship. Questions are about the child's behavior, the parents' reactions and how the family functions.

   [Time Frame: Second follow-up:15 months after baseline (child 9 months old)]

5. Edinburgh Postnatal Depression Scale
   Measuring maternal depressive symptoms. The effectiveness of Edinburgh Postnatal Depression Scale (EPDS) for detection of depression at a clinical level is well-documented, and has been shown to have high sensitivity and specificity against a clinical psychiatric diagnosis of depression.

   [Time Frame: Baseline, first follow-up: 7 months after baseline (child 1 month old) and second follow-up:15 months after baseline (child 9 months old)]

6. Maternal Antenatal Attachment Scale
   Measuring maternal attachment to the unborn child, in terms of thoughts, feelings and behavior towards the unborn child. There are two dimensions: the quality of the attachment (emotional closeness/distance, positive/negative feelings, tenderness/irritation aimed at the unborn child) and intensity/time the mother is in attachment mode (how involved the woman is with the fetus)

   [Time Frame: Baseline]

7. Warwick Edinburg Mental Well-being Scale
   Measuring mental well-being in a broad understanding of the condition. Includes positive emotion, psychological function and interpersonal relations. All questions are formulated in positive phrases.

   [Time Frame: Baseline, first follow-up: 7 months after baseline (child 1 month old) and second follow-up:15 months after baseline (child 9 months old)]

8. Ages and Stages Questionnaire - Social Emotional
   Measuring the infant's socio-emotional development. The mother and father should complete the questionnaire and the responses are assessed separately. Includes self-regulation, compliance, communication, adaptive functioning, autonomy, emotions and interaction with other people.

   [Time Frame: Second follow-up:15 months after baseline (child 9 months old)]

9. Experiences in Close Relationship - revised version
   Measuring adults' attachment in close relationships. Measures 1) avoidant attachment style, characterized by fear of intimacy and interpersonal dependency and 2) fear of attachment, characterized by fear of being rejected and a craving for interpersonal closeness.

   [Time Frame: Baseline, first follow-up: 7 months after baseline (child 1 month old) and second follow-up:15 months after baseline (child 9 months old)]

10. Family and Social Support Scale
    Measuring parents perceived support from family, friends, society and their partner.

    The scale is used in many studies to measure the effect of social support on parents' health and well-being, family integrity, parents' perception of the child and the parent-child interaction.

    [Time Frame: First follow-up: 7 months after baseline (child 1 month old) and second follow-up:15 months after baseline (child 9 months old)]

11. Adverse Childhood Experiences International Questionnaire
    Measuring some of the most intense and frequently occurring causes of stress in the childhood, and the association between these causes and risk behavior later in life. Measuring family dysfunction, physical, sexual and emotional abuse and neglect of care from the parents (or other carers), different types of violence and witnessing of violence.

    [Time Frame: Second follow-up:15 months after baseline (child 9 months old)]

12. Attitude towards breast-feeding

The mothers and the fathers attitude towards breast-feeding before birth – scales to be selected

[Time Frame: Baseline]

13. Breast-feeding status
Self-reported breast-feeding status – scales to be selected

[Time Frame: First follow-up: 7 months after baseline (child 1 month old) and second follow-up:15 months after baseline (child 9 months old)]

14. APGAR-score
Summarizing the health of the newborn child, including skin colour, breath, muscular tension, heartbeat and reflexes. Measured 1, 5 og 10 minutes after birth.

[Time Frame: At birth]

15. Birth weight
Measurement documented in the birth record immediately after birth.

[Time Frame: At birth]

16. Length at birth
Measurement documented in the birth record immediately after birth.

[Time Frame: At birth]

17. Head circumference at birth
Measurement documented in the birth record immediately after birth.

[Time Frame: At birth]

18. Maternal sensitivity
Sensitive responses, that is the ability to respond appropriately to the child's attachment needs, has consistently been found to be the most reliable predictor of attachment security. The intervention, including The Circle of Security, has the aim to promote maternal sensitivity.

The measurement tool "Coding Interactive Behaviour" (CIB) will be used to code maternal sensitivity towards the child. Maternal sensitivity is observed during 5 minutes interaction (free play) between mother and infant.

CIB has good psychometric properties and has been validated in multiple longitudinal studies across cultures and across age groups (from newborn to adolescent). The system has previously been used for the purpose of research of the relation between a child and a mother with mental illness(es). Moreover, it has been used to evaluate the effectiveness of interventions.

[Time Frame: The mother-child relation will be videotaped and assessed during a home-visit by the health visitor when the child is around 1 month.]

Eligibility

Minimum Age: 18 Years

Maximum Age:

Sex: Female

Gender Based:

Accepts Healthy Volunteers: Yes

Criteria: Inclusion Criteria:

- Pregnant women with psychological/psychiatric problems (care level 3*)
- Pregnant women with severe social problems, either economic or familiar (care level 3*)
- Pregnant women from the municipality of Ballerup, Gentofte, Herlev and Rødovre

Exclusion Criteria:

- Pregnant women that exclusively has incident or chronic somatic illnesses (care level 3*)
- Pregnant women with problems related to alcohol, drugs and/or medicine (care level 4*)
- Pregnant women that does not speak/understand the Danish language
- Pregnant women under the age of 18
- The following diagnosis: Active eating disorder, Severe depression , Psychosis, Schizophrenia, Bipolar disorder.

Contacts/Locations

Central Contact Person: Anne Kristine AK Aarestrup
Telephone: (0045)20217269
Email: anne.kristine.aarestrup@regionh.dk

Central Contact Backup:

Study Officials: Ane Friis AF Bendix
Study Director
Intersectoral Research Unit for Health Services

Locations: Denmark
Intersectoral Research Unit for Health Services
Copenhagen, Denmark
Contact: Anne Kristine Aarestrup, PhD 0045 20217269
anne.kristine.aarestrup@regionh.dk
Contact: Michaela L. Schiøtz, PhD 0045 27333083
michaela.louise.schioetz@regionh.dk

References

Citations:

Links:

Study Data/Documents:

U.S. National Library of Medicine | U.S. National Institutes of Health | U.S. Department of Health & Human Services
