# Supplementary material for: Efficacy of a randomized controlled trial of a perinatal adaptation of COS-P in promoting maternal sensitivity and mental wellbeing among women with psychosocial vulnerabilities
Source: PLoS One. 2022 Dec 1;17(12):e0277345. doi: 10.1371/journal.pone.0277345 (PMC9714844; doi:10.1371/journal.pone.0277345)
Supplement: Attachment — Submitted filename: Response to Reviewers.docx [file pone.0277345.s003.docx]

Dear Dr. Palatnik

Thank you for giving us the opportunity to re-submit our manuscript.

We hope the changes made is sufficient and that you will consider accepting the manuscript for publication.

On behalf of the authors,

Katrine Røhder

**General comments:**

1. Please ensure that your manuscript meets PLOS ONE's style requirements, including those for file naming. The PLOS ONE style templates can be found at 
[https://journals.plos.org/plosone/s/file?id=wjVg/PLOSOne_formatting_sample_main_body.pdf](https://eur02.safelinks.protection.outlook.com/?url=https%3A%2F%2Fjournals.plos.org%2Fplosone%2Fs%2Ffile%3Fid%3DwjVg%2FPLOSOne_formatting_sample_main_body.pdf&data=04%7C01%7Ckatrine.rohder%40psy.ku.dk%7C8c1ac6341a504fc2c59208d9d28275f5%7Ca3927f91cda14696af898c9f1ceffa91%7C0%7C0%7C637772280959939051%7CUnknown%7CTWFpbGZsb3d8eyJWIjoiMC4wLjAwMDAiLCJQIjoiV2luMzIiLCJBTiI6Ik1haWwiLCJXVCI6Mn0%3D%7C3000&sdata=nz8TfV3PIjaEzhftV%2Frf9ImowcDesCCd87ycQtquyNs%3D&reserved=0) and 
[https://journals.plos.org/plosone/s/file?id=ba62/PLOSOne_formatting_sample_title_authors_affiliations.pdf](https://eur02.safelinks.protection.outlook.com/?url=https%3A%2F%2Fjournals.plos.org%2Fplosone%2Fs%2Ffile%3Fid%3Dba62%2FPLOSOne_formatting_sample_title_authors_affiliations.pdf&data=04%7C01%7Ckatrine.rohder%40psy.ku.dk%7C8c1ac6341a504fc2c59208d9d28275f5%7Ca3927f91cda14696af898c9f1ceffa91%7C0%7C0%7C637772280959949046%7CUnknown%7CTWFpbGZsb3d8eyJWIjoiMC4wLjAwMDAiLCJQIjoiV2luMzIiLCJBTiI6Ik1haWwiLCJXVCI6Mn0%3D%7C3000&sdata=Kbcc610ju0i1mW7d0nja0kNkQHuNdOq37WrVyKsuE%2Fo%3D&reserved=0)

**Response**

The manuscript and the title page has been formatted according to the templates.

2. Thank you for stating the following in the Acknowledgments/ Funding Section of your manuscript:

The entire project (design, data collection, data analysis, interpretation of data and interpretation of data) is funded by The Capital Region of Copenhagen. A representative from The Capital Region was part of the steering committee of the project. The steering committee support the implementation of the intervention. The steering committee was not involved in data collection, data analysis, interpretation of data, writing the report nor the decision to submit the report for publication.

Please note that funding information should not appear in the Acknowledgments section or other areas of your manuscript. We will only publish funding information present in the Funding Statement section of the online submission form. 
Please remove any funding-related text from the manuscript and let us know how you would like to update your Funding Statement. Currently, your Funding Statement reads as follows:

The entire project (design, data collection, data analysis, interpretation of data and interpretation of data) is funded by The Capital Region of Copenhagen. A representative from The Capital Region was part of the steering committee of the project. The steering committee support the implementation of the intervention. The steering committee was not involved in data collection, data analysis, interpretation of data, writing the report nor the decision to submit the report for publication.

Please include your amended statements within your cover letter; we will change the online submission form on your behalf.

**Response**

We would like the financial statement to be as follows:

“The project was funded by The Capital Region of Copenhagen. The funders had no role in study design, data collection and analysis, decision to publish, or preparation of the manuscript.”

3. In your Data Availability statement, you have not specified where the minimal data set underlying the results described in your manuscript can be found. PLOS defines a study's minimal data set as the underlying data used to reach the conclusions drawn in the manuscript and any additional data required to replicate the reported study findings in their entirety. All PLOS journals require that the minimal data set be made fully available. For more information about our data policy, please see [http://journals.plos.org/plosone/s/data-availability](https://eur02.safelinks.protection.outlook.com/?url=http%3A%2F%2Fjournals.plos.org%2Fplosone%2Fs%2Fdata-availability&data=04%7C01%7Ckatrine.rohder%40psy.ku.dk%7C8c1ac6341a504fc2c59208d9d28275f5%7Ca3927f91cda14696af898c9f1ceffa91%7C0%7C0%7C637772280959959032%7CUnknown%7CTWFpbGZsb3d8eyJWIjoiMC4wLjAwMDAiLCJQIjoiV2luMzIiLCJBTiI6Ik1haWwiLCJXVCI6Mn0%3D%7C3000&sdata=jKrKVXSQB66dkLkWjOe652XBQc7dWoVfU5tOErtX2pM%3D&reserved=0).

Upon re-submitting your revised manuscript, please upload your study’s minimal underlying data set as either Supporting Information files or to a stable, public repository and include the relevant URLs, DOIs, or accession numbers within your revised cover letter. For a list of acceptable repositories, please see [http://journals.plos.org/plosone/s/data-availability#loc-recommended-repositories](https://eur02.safelinks.protection.outlook.com/?url=http%3A%2F%2Fjournals.plos.org%2Fplosone%2Fs%2Fdata-availability%23loc-recommended-repositories&data=04%7C01%7Ckatrine.rohder%40psy.ku.dk%7C8c1ac6341a504fc2c59208d9d28275f5%7Ca3927f91cda14696af898c9f1ceffa91%7C0%7C0%7C637772280959959032%7CUnknown%7CTWFpbGZsb3d8eyJWIjoiMC4wLjAwMDAiLCJQIjoiV2luMzIiLCJBTiI6Ik1haWwiLCJXVCI6Mn0%3D%7C3000&sdata=hI2UBGDP0pi1YLB8lTDENhCK4pPW21i2w5UsZNXnpSU%3D&reserved=0). Any potentially identifying patient information must be fully anonymized.

Important: If there are ethical or legal restrictions to sharing your data publicly, please explain these restrictions in detail. Please see our guidelines for more information on what we consider unacceptable restrictions to publicly sharing data: [http://journals.plos.org/plosone/s/data-availability#loc-unacceptable-data-access-restrictions](https://eur02.safelinks.protection.outlook.com/?url=http%3A%2F%2Fjournals.plos.org%2Fplosone%2Fs%2Fdata-availability%23loc-unacceptable-data-access-restrictions&data=04%7C01%7Ckatrine.rohder%40psy.ku.dk%7C8c1ac6341a504fc2c59208d9d28275f5%7Ca3927f91cda14696af898c9f1ceffa91%7C0%7C0%7C637772280959969026%7CUnknown%7CTWFpbGZsb3d8eyJWIjoiMC4wLjAwMDAiLCJQIjoiV2luMzIiLCJBTiI6Ik1haWwiLCJXVCI6Mn0%3D%7C3000&sdata=icuUvz%2FqwodDhKy2kUx0KkIhxMXL1StDSCwgIBuN6s8%3D&reserved=0). Note that it is not acceptable for the authors to be the sole named individuals responsible for ensuring data access.

We will update your Data Availability statement to reflect the information you provide in your cover letter.

**Response**

The participants in this study have not given consent to make these data publicly available. Therefore, the data and the information regarding the participants cannot be publicly available. A request for access to the data needs approval from appropriate Danish authorities and are subject to Danish regulations on personal data protection. A request for arrangement of data transfer agreements can be sent to the Center for Clinical Research and Prevention, Frederiksberg Hospital, Capital Region of Denmark, Copenhagen, Denmark (e-mail: tfe@regionh.dk) for researchers who meet the criteria for access to confidential data.

4. Your ethics statement should only appear in the Methods section of your manuscript. If your ethics statement is written in any section besides the Methods, please delete it from any other section.

**Response**

The ethics approval has been deleted from the acknowledgements section (lines 453-458). Protocol and registration numbers are updated in the methods section accordingly (lines 126 & 127)

5. Please include captions for your Supporting Information files at the end of your manuscript, and update any in-text citations to match accordingly. Please see our Supporting Information guidelines for more information: [http://journals.plos.org/plosone/s/supporting-information](https://eur02.safelinks.protection.outlook.com/?url=http%3A%2F%2Fjournals.plos.org%2Fplosone%2Fs%2Fsupporting-information&data=04%7C01%7Ckatrine.rohder%40psy.ku.dk%7C8c1ac6341a504fc2c59208d9d28275f5%7Ca3927f91cda14696af898c9f1ceffa91%7C0%7C0%7C637772280959969026%7CUnknown%7CTWFpbGZsb3d8eyJWIjoiMC4wLjAwMDAiLCJQIjoiV2luMzIiLCJBTiI6Ik1haWwiLCJXVCI6Mn0%3D%7C3000&sdata=V5cZxD1aqrAAWo47E2y3HqwVhxOodIvUl7ioAOFBSos%3D&reserved=0).

**Response**

We do not have any supporting information files.

**Responses to Questions from Reviewer 1:**

**Abstract**

- typo in line 20: 'woman' instead of 'women'.

- typo in line 28: 'midwifes' instead of 'midwives' - and in the rest of the manuscript, e.g. line 107

**Response**

Thank you, it´s corrected throughout the manuscript.

- the results paragraph of the abstract should include some numerical results, specifically the estimates of effect for key outcomes with 95% confidence intervals and p-values; perhaps including a sentence on the total number of women recruited.

**Response**

Information on the total number of women recruited is included in line 31. Furthermore, we have added regression coefficients, 95% CI and p-values for the primary outcome maternal sensitivity and parental stress, total scale. The wording is as follows (lines 38-42):

*“We did not find an effect of the intervention on the primary outcome of maternal sensitivity (β = -0.08; 95% CI [-0.41, 0.26], p =.66). Neither did we find intervention effects on the secondary outcomes of depressive symptoms, parental reflective functioning, maternal well-being, or infant socio-emotional functioning. We did however find that the intervention decreased parental stress (β = -8.51; 95% CI [-16.6;-0.41], p = .04).”*

**Background**

- line 74: please report the magnitudes of the 'medium effect sizes' mentioned here.

**Response**

The information on effect sizes and p-values have been added in lines 80-82. The text is a follows:

*“A recent meta-analysis of the effect of the COS and COS-P intervention (18) found medium effect sizes for improving child attachment security (Hedge´s g = 0.65, p = .003), quality of caregiving (Hedge´s g = 0.60, p = .012), and reduction of caregiver depression (Hedge´s g = 0.53, p < .001), and a large effect size for improving caregiver self-efficacy (Hedge´s g = 0.98, p < .001).”*

line 80: please state objectively what the 'promising results' were.

**Response**:

A more detailed account of the results from the Cassidy et al (2010) study is given in lines 87-92. The text is now as follows:

*“This study found promising results with infant attachment status and maternal sensitivity resembling low risk groups. After the intervention, 70% of the study children were securely attached while 20 % of them were classified as insecure-disorganized. There was no difference in maternal sensitivity between mothers participating in COS (M = 8.55, sd = 2.42) and mothers from a community sample (M = 7.33, sd = 2.26) in a 10-minutes free play interaction. The study did not include a control group which limits the conclusions that can be made from this study.”*

**Methods**

- Procedures - should be consistently reported in the past tense like the rest of the manuscript. The beginning is reported in past tense, but sections such as line 130-135 are in the present tense.

**Response**

This has been corrected.

- Intervention - the 'perinatal adaptions' to the COS-P intervention mentioned in line 141 should be clearly described.

**Response**

The perinatal adaptation is described in the following lines 156-157 and consisted only of a change in the order of which the chapters are presented to mothers. To avoid confusion the following sentence has been added in line 154-155:

*“The adaptation consisted of an alteration of the order of when chapters were presented to the parents.”*

- the CIB outcome should be described in a more detail. It is not clear how it is scored (if it is in fact a score), what is the measurement scale, what are the normal ranges, does it have any components/items/domains and if so how are they scored, etc. The same detail should be included for the secondary outcomes.

**Response**

All outcomes have been updated to describe this information.

Revision regarding the CIB is as follows (lines 162-170):

*“The CIB is a global rating scheme for coding interactions between parent and infants aged 2 to 36 months. The CIB at nine months consists of 33 items that are aggregated into composites of maternal sensitivity and intrusiveness, infant involvement and withdrawal, dyadic reciprocity, and dyadic negative states. Each items is coded on a 5-point scale with high scores representing many observations of the given behavior or affective state being rated. There are no norms for the CIB. The CIB has been used to detect improvements in parental behavior following perinatal interventions (25, 26). The current study used the maternal sensitivity composite.”*

Additional information on the ASQ-SE has been added in lines 188-191, on the PRF lines 196-203, the PSI-SF lines 207-210, the EPDS lines 214-216 and the SWEMWBS lines 220-222.

- statements in lines 152-153 about the Cronbach's alpha and in 158 about the ICC are results and should be reported in the results section.

**Response**

The information on cronbach´s alpha and ICC are removed to the results section, lines 311-315.

- the sample size is not well described, partly because the primary outcome is not well described either. If the primary outcome is continuous, then the sample size calculation should include some idea about what the expected mean and SD of the outcome in the control group (plus the difference to be detected). You should also state whether the level of significance is for a one-sided or two-sided test.

**Response**

The section on power analysis has been updated with the following paragraph (lines 227-236):

*“Power analysis was conducted prior to recruitment and described in our protocol paper (22). The CIB uses an interval scale from 1 to 5. We assumed that the average mean of CIB-maternal sensitivity would be 3 with a standard deviation of 0.9. One study found a difference of 0.9 between mothers with and without depression. We expected a between-group difference of 0.75. Based on a t-test, 80% power and a two-sided significance level α of 0.05and therefore waimed to include 48 families with 24 per group. We estimated a likely dropout rate of 20% and therefore aimed to include 60 families (30 in each group) at randomization.“*

- as this is a randomised trial, you should not conduct tests comparing descriptive characteristics between the groups (line 197-199).

**Response**

We agree and have deleted the tests and descriptions thereof (lines 239-241).

- for the same reason above, any adjustment in the analysis should not normally be based on whether there are differences in baseline characteristics; however, design factors such as the stratification variables should be adjusted for.

**Response**

After conducting intention-to-treat analysis on an imputed dataset (results reported), we conducted a number of adjusted analysis. Of these, we conducted an analysis, where we controlled for background variables, which we hypothesized could impact the outcome. We didn´t conduct analysis due to group differences even though we agree that that is not clear from the manuscript. Therefore, we changed the wording (lines 245-247). It now says:

*“Secondary, the analysis was adjusted for background variables (parity, education, maternal age, baseline parental measures) due to prior hypothesis regarding their impact on the outcome and analyzed using the per protocol principle.”*

Furthermore, we conducted new analysis to control for stratified study design. This did not change the results. We added a new sentence describing this analysis (lines 252-255):

*“Both multiple imputation and regression analyses were with municipality included due to the stratified design of the study. Municipality was included/modelled as a random effect in mixed models.”*

**Results**

- given my comments above regarding analysis of a randomised trial, the results on line 233-234 about between group differences, and the p-value column on Table 1 (and the footnote) should be removed.

**Response**

As indicated above, we agree and have deleted lines 279-280 and the p-value column in Table 1 incl. footnote.

- Table 1 should report means and standard deviations (SD) for continuous characteristics. It is unclear whether the quantities reported against EPDS are the mean and standard deviation. Table 1 should also be clear when reporting counts and proportions of categorical variables, and explicitly state this.

**Response**

Thank you for pointing that out. The information has been added to the Table.

- Table 2 should report means and standard errors (SE) for continuous outcomes. The 'range' column is not necessary here, and should have been included in the description of the measures (see comments above)

**Response**

Thank you. The reported means and standard errors are indeed standard errors and not standard deviations. We made sure that this has been corrected. The range column has been deleted and the information added to the Methods section.

- again in Table 2, for outcomes with domains/components, p-values should generally be reported for the total or overall score, not the components, as this constitutes multiple testing which then warrants corrections that are difficult do to in this small study.

**Response**

Only the PSI-SF has subscales. We have deleted testing among these subscales and now only report group differences among overall scores, which is in line with the study aims and described in the protocol paper.

- for outcome measures where you have baseline values, instead of conducting an analysis on the change-from-baseline measure, you should ideally conduct an analysis on the post-intervention measures and adjust for the baseline measures (i.e. analysis of covariance ANCOVA). This is the most statistically-efficient way to analyse these outcome.

Response

Thank you for this comment.

This is indeed what we did which is stated in the Method section lines 255-257. We changed the wording in the footnote to be more precise.
