# Supplementary material for: Efficacy of a randomized controlled trial of a perinatal adaptation of COS-P in promoting maternal sensitivity and mental wellbeing among women with psychosocial vulnerabilities
Source: PLoS One. 2022 Dec 1;17(12):e0277345. doi: 10.1371/journal.pone.0277345 (PMC9714844; doi:10.1371/journal.pone.0277345)
Supplement: Attachment — Submitted filename: Response to Reviewers.docx [file pone.0277345.s004.docx]

To reviewers and editor:

Thank you both for your comments and for the opportunity to re-submit our manuscript. Based on these, we have made additional changes to the manuscript to encompass your reflections. Our replies to your comments are highlighted in blue. We refer to changes made in the manuscript (lines) *with* tracked changes.

1. **Reviewer #1:** In line 274 you have stated that the means and standard deviations are reported in Table 2. However the column titles of Table 2 appear to indicate that you are reporting standard errors (which is the correct thing to report here, as this is a table of inferential results). So either (1) correct the statement on line 274 or (2) report standard errors in Table 2 if they are in fact standard deviations and correct the statement on line 274.

Thank you for spotting this. We are reporting standard errors in Table 2. We have corrected the statement on line 327.

**Reviewer #2:** The paper is a randomized controlled trial exploring the efficacy of the COS-P intervention, adapted to the perinatal period, in improving several aspects of mothers’ mental health and well-being. Despite the topic is interesting, there are several aspects that should be clarified, before resubmitting the paper:

1. The primary aim of the study is to increase of “maternal sensitivity in interactions with the infant nine months after birth”. The concept of “maternal sensitivity” is not really clear to me. No explanation of this is provided in the introduction and/or in the method section. Could the authors better specify what they intend for “maternal sensitivity”. Similarly, among secondary outcome it is reported that the intervention could lead to a “better maternal ability to mentalize”; could this issue be better described?

We added a section in the introduction on maternal sensitivity and parental reflective functioning (lines 60-71). Furthermore, we decided to use the wording “parental reflective functioning” instead of “mentalizing abilities” throughout the manuscript as this is more consistent and in line with the measure used (the P-PRFQ).

*“Early parenting behavior is considered the foundation for child development, with numerous studies demonstrating that “sensitivity” is one of the strongest predictors of positive developmental outcomes for children across different domains (11-14). Sensitivity has been defined by Ainsworth and colleagues as a parent´s ability to 1) notice child signals, 2) interpret these signals correctly, and 3) respond promptly and appropriately to these signals (15). Parental mental health problems are associated with an increased risk of lower sensitivity (16, 17) with sensitivity mediating the association between antenatal mental health problems and behavioral problems in the child (18, 19). A related concept is parental reflective functioning which is defined as the parent's capacity to reflect upon her child's emotional experience and her own experiences as a parent (20). Maternal reflective functioning capacities has been linked to caregiving behavior (e.g. sensitivity) and studies have demonstrated that higher levels of reflective functioning are associated with secure child attachment, better emotion regulation abilities, and less psychopathological symptoms (21).”*

2. One of the inclusion criteria is that mothers had to present “psychosocial or social vulnerabilities” identified by the general practitioner. This is too vague and undefined. Please specify in detail the inclusion criteria, since this is a RCT and inclusion criteria are crucial to select the right population to be treated.

The inclusion criteria were made based on the official Danish Health Care recommendations. This was both a clinical and practical decision. Clinically, it was considered an advantage that potential participants were already identified and assessed (based on clinical records, self-reported information from pregnant women, and clinical presentation) by clinical staff as “being in risk” and in need of additional interventions. Practically, this group was referred to a special unit at the hospital where a smaller group of midwifes could be trained to recruit potential participants.

We have made the Participant section more clear with a headline and included the following paragraph in the manuscript (lines 133-136):

*“This is in line with official Danish Health Care recommendations (34). The group consists of women with complex psychological/psychiatric problems and/or difficult social problems with a need for interdisciplinary perinatal care.”*

3. The experimental intervention is poorly described. Authors should explain in details the experimental intervention (i.e., how many sessions were provided to mothers, what was the content of sessions, how session were structured, etc.), and report the appropriate citation to the available manuals.

We inserted a reference to the manual and a table (Table 1; line 185) describing number, content and time point of sessions. The reference can found at line 179 in the manuscript.

4. The main outcome was assessed through the CIB. Is this global rating scheme validated? Were raters trained in the use of the assessment instruments? Moreover, authors should specify whether mental health professionals who performed the assessments were blinded to mothers’ allocation to the experimental vs. control group.

More information on the CIB has been added to the Method section (lines 194-201) :

*“The CIB has been validated in both longitudinal and intervention studies showing predictive validity for child development outcomes (37, 38), discriminate validity between mothers with and without mental health problems (17), as well as ability to detect effects of perinatal interventions (39, 40). The current study used the maternal sensitivity composite. At approximately nine months, mother-infant interactions were videotaped during a home visit by a researcher not blinded to mothers’ allocation to the experimental vs. control group. A coder trained in the CIB by Ruth Feldman blinded to treatment allocation coded all mother-infant interactions.”*

5. I was not able to find thought the manuscript when women were assessed for the first time (I suppose during pregnancy) and when they were reassessed. Could the author better specify this issue?

Information on the procedure and data collection has been added to the manuscript (lines 146-151):

*“A researcher contacted eligible participants that consented to be contacted by phone, followed by a home visit to provide further information on the project, obtain informed consent, and enroll interested women and their families in the project. Data collection took place at three time points: at study inclusion at 3–5 months of pregnancy, when the infant is 8 weeks old, and after the intervention has ended when the child is 9 months old. Only data from the baseline assessment and after the intervention are reported in this study.”*

6. In the result section, it is reported that 35 out of 76 women presented mental health problems at pregnancy and 28 of them were on psychiatric or psychological treatment. This is a bit confusing, since authors have excluded from the recruitment women with major psychiatric problems. Could you clarify this issue?

As specified in the Participant section, inclusion criteria were pregnant women experiencing complex psychological difficulties and/or psychiatric disorders, such as mild to moderate depression, anxiety, personality disorders and/or ADHD. Exclusion criteria were *specific* psychiatric disorders (active eating disorder, severe depression, psychosis, schizophrenia, bipolar disorder, and severe obsessive-compulsive disorder) as women with these disorderes already received treatment elsewhere. We did not expect women with such severe disorders to be able to participate in two interventions due to limited mental and practical resources.
